# A novel approach to the treatment of acuterenal failure associated with rhabdomyolysis

**DOI:** 10.1186/2197-425X-3-S1-A61

**Published:** 2015-10-01

**Authors:** T Laddomada, A Doronzio, B Balicco

**Affiliations:** Policlinico San Marco, Anesthesia and Intensive Care Service, Zingonia, Italy

## Introduction

Acute renal failure (ARF) is a common complication of Rhabdomyolysis (RBM), a syndrome characterized by muscle breakdown and necrosis resulting in the leakage of the intracellular constituents into the circulation and the extracellular fluid. It is estimated that about 10-50% of patients with RBM develop ARF.

## Objectives

All the classic treatments for RBM have demonstrated only partial efficacy. Intravascular volume expansion, urinary alkalinization, and forced diuresis are currently used as renal-protective measures but are not useful in the context of severe oliguria. Acute kidney failure is treated by classical renal replacement therapies; it's important to produce also a significant removal of circulating myoglobin and the use of blood purification techniques may be advantageous. Literature has shown that previous attempts to remove myoglobin using plasma exchange, intermittent hemodialysis, and continuous renal replacement therapies (CRRT) have unfortunately met with limited success. With an analysis of the literature, we aimed to evaluate a new solution for the removal of myoglobin in the treatment of RBM, represented by a dedicated sorbent that can be used both in hemoperfusion (HP) and in combination with traditional CRRT.

## Methods

Literature provided a published case study in which a new adsorber, CytoSorb^TM^ (CytoSorbents Corp) was used in HP for 12h to remove myoglobin from blood in a complex situation of legionella-pneumonia associated RBM. CytoSorb^TM^ is a sorbent made of an advanced biocompatible porous polymer with high binding capacity and designed to reduce toxic levels of pro- and anti-inflammatory mediators, myoglobin and bilirubin directly from blood. The evidence found in literature is supported by our clinical experience, not published yet, regarding a patient with severe RBM and ARF, after laparoscopic sleeve gastrectomy. The cartridge was used for 24h and it was installed in a series connection after the dialyzer into the CRRT circuit.

## Results

The course of standard laboratory makers and myoglobin levels are shown in the table. Results demonstrated an important decrease of myoglobin levels and an improvement in renal function both in stand-alone mode and in combination with CRRT.

**Table 1:** Results before and after the treatment.

	HP	CRRT + HP				
	T0	T1	Reduction	T0	T1	Reduction
**Creatinine (mg/dL)**	1,56	1,15	26,3%	3,4	2,1	38,2%
**Myoglobin (ng/mL)**	18390	8359	54,6%	52998	8862	83,3%
**Creatine Kinase (U/L)**	-	-	-	18587	8755	52,9%

## Conclusions

The use of new sorbents in continuous veno-venous filtration and HP might represent a novel approach to the treatment of acute RBM not only because efficient renal replacement may be provide but also because a potential protective effect can be envisaged in the rapid and efficient removal of circulating myoglobin and creatine kinase.
